# An ML-based Framework for Early Cerebral Stroke Prediction using Clinical Data

**DOI:** 10.2174/0115734056466362260527062043

**Published:** 2026-09-22

**Authors:** Asma Aldrees

**Affiliations:** 1 Department of Informatics and Computer Systems, College of Computer Science, King Khalid University, Abha, 62521, Saudi Arabia

**Keywords:** Cerebral stroke prediction, SMOTE, Clinical features, Extra trees classifier, Precision medicine, (AI)-based framework, Global health challenge

## Abstract

**Introduction:**

Cerebral stroke remains a major global health challenge, where early and accurate diagnosis is critical for reducing mortality and long-term disability. The challenges, such as missing data, class imbalance, and increasing complexity of clinical variables, limit the effectiveness of conventional diagnostic approaches. This study aims to develop an Artificial Intelligence (AI)-based framework for early, accurate, and explainable cerebral stroke prediction using structured clinical data.

**Methods:**

This research proposes an ML-based framework that integrates clinical/tabular data for early prediction of cerebral stroke. The framework employed a Multi-layer Extra Trees Classifier (ETC) incorporating mean imputation and KNN imputation techniques, which are applied for handling missing values, while the Synthetic Minority Over-sampling technique (SMOTE) is used for class imbalance in clinical data. Experimental results demonstrate that the ETC achieves higher performance for early stroke risk assessment and improving decision-making in healthcare settings.

**Results:**

Experimental evaluation demonstrates that the proposed ETC combined with mean imputation and SMOTE significantly outperforms baseline models. The proposed model achieves an accuracy of 99.25%, precision of 98.85%, and recall of 98.92%, highlighting its robustness and effectiveness in early stroke prediction using clinical data.

**Discussion:**

The results indicate that appropriate data preprocessing techniques like mean imputation and SMOTE data balancing lead to substantial performance gains. The ETC shows predictive capability in capturing patterns in tabular clinical data. These findings support early stroke risk assessment and assist in decision-making in healthcare.

**Conclusion:**

Overall, the proposed framework advances early cerebral stroke diagnosis using structured clinical data. By achieving high predictive performance, this work provides a strong foundation for AI-assisted stroke prediction using tabular healthcare data and contributes to preserving clinical decision support systems.

## 1 INTRODUCTION AND BACKGROUND

Stroke is a leading concern worldwide because it is among the primary diseases leading to mortality and disability, ranked at the top second in deaths worldwide. Both genders are >equally susceptible to it, hampering their life quality and putting pressure on the healthcare system. Over the last three decades, the incidence of strokes has grown by 70%, deaths by 43%, and disability adjusted life years (DALY) by 32% [1, 2]. This rise is mainly attributed to changing stroke patterns in low- and middle-income countries (LMICs). It is anticipated that by 2030, stroke will cost over 200 million annually as DALYs, contributing 12 million deaths alongside 70 million elderly patients [3]. This growing burden places significant pressure on the healthcare system and highlights the urgent need for effective early prediction and intervention strategies.

Clinically, stroke is caused by clogged blood vessels in the brain or a brain hemorrhage, disrupting the brain’s blood flow and leading to cell degradation due to oxygen and nutrient deprivation. Oxygen-supplying blood vessels to the brain are affected by stroke in a cerebrovascular condition, potentially causing rapid cell death, and without prompt and proper treatment, it can result in severe disabilities [4,5]. The chances of cerebral stroke occurrence depend directly on your behavioral patterns, lifestyle choices, sociocultural factors, and demographic shifts such as aging. A stroke can either be hemorrhagic or ischemic [6]. In a hemorrhagic stroke, a less common type, blood vessels rupture, resulting in bleeding in the brain. The most predominant type of cerebral stroke is ischemic, which occurs when an artery is blocked or narrowed, hindering the blood supply to a certain brain part. Stroke symptoms vary and may include neurological impairments; however, early prediction of stroke risk remains a critical challenge in clinical practice [7].

In recent years, Artificial intelligence (AI) and machine learning (ML) have shown significant potential in addressing clinical diagnosis issues. Deployment of various AI-based tools for risk assessment of stroke-related emergencies offers promising avenues of exploration in this domain [8]. AI techniques and data related to brain strokes can significantly contribute to medical research by providing immediate responses about the cause of stroke, stroke patterns, and other risk factors. Based on these things, AI develops treatment plans to help health practitioners. Moreover, stroke data obtained from observational studies and clinical trials can improve the understanding of long-term outcomes and quality of life for stroke survivors, creating space for further research improvements. Several studies have explored the application of ML and AI techniques for cerebral stroke prediction. Traditional approaches have demonstrated promising results using clinical datasets. Tazin *et al*. utilized various physiological parameters and ML algorithms (RF, DT, voting classifier (VC), and LR) to construct four distinct models for accurate prediction [9]. They utilized a publicly available Kaggle cerebral stroke prediction dataset for model development. In particular, RF shows top performance, achieving an accuracy of approximately 96%. Dritsas and Trigka utilized ML approaches to explore early stroke detection, a critical need given that the sudden abruption in brain blood flow potentially leads to impairment or fatality [10]. This research also utilized multiple ML models (RF, Naive Bayes (NB), LR, KNN, and stacking) and techniques to provide the most effective solution. Their datasets comprise 3254 individuals aged 18 and above. The stacking technique yielded impressive results, achieving 98% accuracy, 97.4% precision-recall, and 98.9% area under the curve (AUC).

Harshitha *et al*. performed a study to identify the possibility of stroke through five different ML techniques: RF, LR, SVM, and KNN [11]. Among these models, RF and LR had the best accuracy, which was at 95.5% of overall accuracy, resulting in a selection of these models as the most accurate and precise, minimizing the cases of false negatives. Maridha *et al*. developed an automated system for stroke prediction; the study used six different ML models [12]. In practice, they assessed the performance of the developed ML technique by examining its generalization capability as well as the prediction accuracy. Moreover, to give the audience a brief understanding of how ML works, they explained two types of explainable techniques, including SHAP and LIME. It emerged from the findings that the RF prototype was superior to the other learning models, delivering an accuracy of 90.36%.

Shinde *et al*. proposed an ML model for the early prognosis of stroke [13]. The primary focus of the study was to advance and evaluate various ML models for the prediction of cerebral stroke probability. Through a comprehensive experimental assessment utilizing Synthetic Minority Over-sampling Technique (SMOTE) and SMOTE ENN for class balancing, the stacking classification approach demonstrated remarkable performance, achieving an accuracy of 99.52% using SMOTE ENN. Similarly, Mia *et al*. investigated the use of strong ML algorithms, including LR, RF, and KNN, for forecasting cerebral strokes [14]. The Harvard Dataverse Repository is used to collect stroke-related data. For class balancing, three different methods were employed: adaptive synthetic sampling (ADASYN), SMOTE, and the random oversampling method. The study’s results indicated that RF, coupled with the ADASYN oversampling technique, achieved an accuracy score of 99%.

Choi *et al*. introduced a stroke prediction system based on ML [15]. Their study began by comprehensively understanding the roles of variables in stroke modeling. Subsequently, they evaluated the predictive performance of decision trees. The results indicated that all predictive variables, except BMI, significantly influenced stroke occurrence. Overall, DT achieved the highest accuracy score of 98.17%. Ray *et al*. proposed a cloud-based platform for stroke disease prediction [16]. They employed SMOTE for class balancing and Chi-square for feature extraction. Their study utilized the cloud-based platform Microsoft Azure ML (AzureML). Results revealed that the boosted decision trees (DT) with six features attained the highest accuracy score of 96.8% on the SMOTE balanced dataset.

Liu *et al*. introduced a mixed ML method for forecasting brain stroke in medical diagnosis using physiological data with class imbalance and incompleteness [17]. They deployed a two-step approach in their study. The first step is the imputation of missing values using random forest regression before classification. The second step involves forecasting for stroke on an imbalanced dataset with the deployment of DNN-based automated hyper-parameter optimization (AutoHPO). The highest accuracy rate of 71.6% was reported by the system. Kokkotis *et al*. focused on developing dependable ML-based forecasting prototypes for stroke [18]. Their study utilized class balancing methods to address class imbalance issues and employed SHAP for interpreting the model output to understand the decision-making mechanism. They conducted a comparative analysis of the anticipated ML method with six eminent classifiers regarding metrics related to prediction accuracy and generalization capability. The results revealed that LR attained the highest accuracy score of 73.52%. Jing proposed an ML system to envisage stroke based on imbalanced medical datasets [19]. They investigated potential risk factors for stroke and introduced a deep learning system utilizing PCA-Kmeans + Focal Loss. Results demonstrated that the recommended method accomplished the highest accuracy score of 92%.

When we summarized the literature review, we found that there is a gap available to address the deadliest cerebral stroke prediction by handling class imbalance and missing values problems. It is evident from previous state-of-the-art research works that the utilization of data resampling and data pre-processing (normalization, imputation) techniques significantly improves the accuracy of models. An overview of the aforementioned literature is provided in Table **1**.

### 1.1 Problem Statement

Although artificial intelligence has been developed to a great extent in medical diagnosis, early detection of cerebral stroke is still an unresolved problem due to the existence of missing data, imbalance in classes, and variability in clinical features. Most of the existing research is based on tabular clinical data, and insufficient handling of data issues limits their predictive effectiveness and generalizability.

The hypothesis in this research is that an efficient ML-based predictive model, with the help of useful data-preprocessing methods such as imputation and class balancing, can lead to a much higher level of accuracy and reliability of early stroke prediction. Thus, the purpose of this work is to design and evaluate a reliable prediction framework, which will solve the issue of data quality and allow for predicting stroke risk more reasonably and accurately using clinical data.

### 1.2 Objectives

Referring to the problem mentioned at the start of the introduction section and in the problem statement, the objective of this study is to enhance the early prediction of cerebral stroke and support clinical decision-making using ML techniques. The proposed approach involved the utilization of nine ML models, including decision tree (DT), logistic regression (LR), random forest (RF), extra tree classifier (ETC), K nearest neighbor (KNN), stochastic gradient descent (SGD), and support vector machine (SVM). The class imbalance issue is addressed by applying the Synthetic Minority Oversampling Technique (SMOTE). This study makes several contributions to the literature in the following areas:

- This study proposes a predictive modeling framework for the early detection of cerebral stroke, employing mean value imputation combined with SMOTE-based data upsampling and an Extra Trees Classifier to improve classification accuracy and reliability.
- A series of comprehensive experiments was conducted to evaluate the effectiveness of the proposed methodology under different data preprocessing strategies, including removal of missing values, mean imputation, KNN imputation, KNN imputation with SMOTE, and mean imputation with SMOTE.
- Performance comparison of the extra tree classifier model and multiple other learning models, including DT, KNN, SVM, LR, RF, and SGD.
- Validated the proposed approach by comparing its performance against baseline ML models, employing k-fold cross-validation to assess its robustness and generalizability.

The remainder of the paper is structured as follows: Section 2, named “Materials and Methods” details the dataset, including preprocessing techniques and data visualization aimed at uncovering hidden patterns. This section also discusses the various algorithms employed in our research. Section 3, called “Experimental Results,” presents a thorough analysis and discussion of the results. Section 4 ”Discussion with Advanced Approaches from Literature” gives insights into how this research work is better than previous research works conducted in this domain. The ”conclusion” section concludes the results, basic themes of this research work, and future work directions.

## 2 MATERIALS AND METHODS

In this section, we detail the methodology employed to conduct the experiments and evaluate the proposed approach for predicting cerebral brain stroke in its early stages. We describe the dataset used for training and testing the models, the preprocessing steps applied to the data, and the implementation of the extra tree classifier model. Additionally, we outline the experimental setup, including the selection of performance metrics, cross-validation procedures, and baseline models for comparison. The following subsections provide comprehensive insights into each aspect of the methodology, elucidating the steps taken to ensure the rigor and reproducibility of the study. The complete proposed methodology is shown in Fig. (**1**).

### 2.1 Dataset

In this research work, we have utilized a publicly available Kaggle dataset named Cerebral Stroke Prediction-Imbalanced Dataset [20]. The dataset includes patients' history, vitals, and 10 other features that are involved in the prediction of cerebral stroke. The dataset contains 43400 patient records. The dataset includes patients ranging in age from approximately 1 month to 82 years, where 0.08 years represents the youngest recorded age. The gender distribution is 25,665 females, 17,724 males, and 11 records of other. Further details about the dataset are provided in Table **2**.

### 2.2 Data Preprocessing

Data preprocessing plays a key role in model reliability and the accuracy of predictive models. In data preprocessing, multiple methods, including data cleansing, imputation, and resampling techniques, will be used [21].

In this research, the missing values problem is resolved by imputing them with the mean of other available values. The second step of pre-processing is dataset class-imbalance, as this dataset contains 42,617 records belonging to the “no stroke/normal” class and 783 records belonging to the “stroke” class. This highly imbalanced dataset problem is resolved by the SMOTE upsampling method.

• Eliminating the missing values from the dataset.
• Imputing similar instances based on missing values using the KNN imputer.

After preprocessing and feature engineering, the final dataset consisted of 11 input features derived from the clinical variables. Categorical features were transformed using label encoding, and continuous features were normalized using z-score standardization. The dataset was divided into training and testing sets using a 70:30 ratio, and a fixed random seed (42) was used to ensure reproducibility. Furthermore, 5-fold cross-validation was applied on the training set to evaluate model robustness and generalizability. The model performance was assessed using accuracy, precision, recall, and F1-score, with particular emphasis on recall and F1-score due to the imbalanced nature of the dataset.

#### 2.2.1 Removing Missing Values from the Cerebral Stroke Dataset

Missing values in the dataset are addressed by eliminating such value-containing instances. We selected to explore this missing-values field-elimination technique in the initial experiment.

#### 2.2.2 KNN Imputer

The KNN imputer is a valuable tool in ML for handling missing values in datasets [22]. It functions by estimating missing values based on the characteristics of neighboring data points. This technique is effective for imputing missing values in both numerical and categorical data. By considering the k-closest training examples in the feature space, the KNN algorithm can be applied to classification or regression tasks. In classification, the most common class among its neighbors is assigned to the object, while in k-nearest neighbors, the average values are the output in the regression.

Overall, the KNN imputer serves as a robust solution for managing missing data within datasets, offering a more advanced approach compared to basic methods like filling missing values with mean or median values. This research work data imputation details are shared in Table **3**.

### 2.3 Class Balancing

Due to the highly imbalanced nature of the dataset, with one class significantly outnumbering the other, namely 42,617 instances belonging to the “no stroke” class and only 783 instances belonging to the “stroke” class, the performance of learning models is notably impacted. To address this issue, we employed SMOTE as a class-balancing method.

#### 2.3.1 SMOTE

Before using SMOTE, the dataset likely had a significant class imbalance, with a much larger number of instances belonging to the majority class (*e.g*., ”no stroke” in this case) compared to the minority class (*e.g*., “stroke”). After applying SMOTE, synthetic data points were generated for the minority class to balance the dataset [23]. This resulted in a new training dataset where both classes are more evenly represented. Before using SMOTE:

• Majority class (*e.g*., “no stroke”): 42,617 instances
• Minority class (*e.g*., “stroke”): 783 instances. After using SMOTE
• Synthetic data points increase the quantity of instances in the minority class.
• Both classes are more balanced, which can help improve the performance of ML models, especially in clinical applications where class imbalance is common and crucial for accurate predictions.

The exact number of instances in each class after using SMOTE would depend on the specific parameters used for the SMOTE algorithm and the desired level of balance in the dataset.

### 2.4 ML Models

The supervised learning method is involved in ML classification, where designated datasets are used to train the system, and new data points are categorized using this information. Regression and classification tasks are addressed by commonly deploying these algorithms. In the subsequent section, we delve into the utilization of ML classifiers for detecting cerebral strokes. The implementation of ML models is facilitated through the scikit-learn library. Seven distinct ML algorithms, namely DT, SVM, ETC, KNN, RF, LR, and SGD, are employed. All models are developed within the Python environment using the scikit module.

#### 2.4.1 Decision Tree

A tree-based classifier, DT, is deployed for classification tasks. Both the regression and classification problems are efficiently handled by it [24]. The primary challenge associated with DT is the root node determination involving attribute selection at each level. Attribute selection techniques such as the Gini Index (GI) and information gain are commonly employed for this purpose. The Gini Index is utilized to compute the Gini value, representing the dataset's impurity. Attribute selection is done using both the Gini index and the information gain, while trees are constructed across all tree-based classifiers in this paper.

#### 2.4.2 Random Forest

Within the ML domain, RF is applied for both the regression and classification tasks, being the greatly established ensemble learning method [24]. It operates as a combination method for decision trees, wherein multiple decision trees are created, and their outputs are amalgamated to yield the eventual forecast. The fundamental philosophy involved in this method is the training of various decision trees based on distinct data subsets for each DT, and then their estimates are merged together to arrive at the ultimate prediction. This method mitigates the overfitting risk associated with training a solo DT.

#### 2.4.3 Extra Tree Classifier

The ETC aims at achieving precise predictions being the multiple DTs with an ensemble learning algorithm [25]. In contrast to RF, ETC adopts a different strategy by randomly selecting an optimal feature subset and then deploying it within the DT at each split. This results in decorrelated tree generation where individual feature sensitivity is minimized, as illustrated by each tree, rendering them more noise-resilient. For the best feature selection criterion, ETC uses the Gini index to segregate the data and assesses the Gini score based on the features’ importance.

#### 2.4.4 K-Nearest Neighbors

The KNN algorithm operates as a non-parametric method that relies on identifying the nearest neighbors to the point under prediction [26]. For instance, within a dataset represented by the training set *x*, the algorithm locates all neighbors of *x*. As there may exist overlaps among these neighbors, the algorithm assigns a score to the *k* neighbors, considering only those with the highest scores.

#### 2.4.5 Logistic Regression

Being the statistical model, LR is employed for binary classification [27]. It finds common applications such as forecasting whether a patient has a disease, discerning spam emails,* etc*. The probability of certain input-based binary results is estimated using LR, and thereafter, employing this estimate to make forecasts. The logistic or sigmoid function is employed to model the likelihood of a binary conclusion. The output is then utilized for prediction. LR can be applied to multi-class classification besides binary classification problems, where more than two classes are involved. For multi- class classification, LR can utilize either the one-vs-all or SoftMax regression approach.

#### 2.4.6 SVM

SVM is a model with the prime objective of a distinguished boundary line within a higher-dimensional space, with N features-defined datasets [26]. These boundaries can be defined by using various hyperplanes, yet the main focus is on margin maximization using pinpointed hyperplanes, suggesting the maximum possible distance between data points of discrete classes. This arrangement boosts confidence in classifying future measurements accurately. The SVM procedure creates a hyperplane in an infinite-dimensional space, supporting numerous activities like feature extraction, data categorization, filtering, and regression. The hyperplane is of prime importance and produces a superior solution based on maximized distance to the closest training instance of any category.

#### 2.4.7 SGD

SGD is a widely acclaimed optimization technique that iteratively learns the optimal values of a model’s parameters to minimize the cost function (*c^f^*). SGD, a notable variant of gradient descent (GD), introduces randomness by selecting a single sample for training in each iteration [28]. This stochastic approach significantly reduces training time, as SGD focuses on the cost function of individual training samples at each iteration to converge towards local minima. Consequently, SGD updates the model parameters for each iteration based on the sample and its corresponding target class.

### 2.5 Evaluation Parameters

In this study, Recall, F1 score, Accuracy, and Precision are used as evaluation metrics to assess the effectiveness of the cerebral stroke prediction model. These evaluation metrics are derived from the confusion matrix. Mathematically, these can be computed as mentioned in eq (**1**):


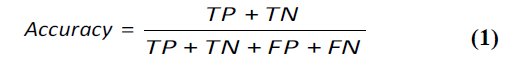


Precision quantifies the ratio of TP among all predicted positives. It is defined as mentioned in eq. (**2**).


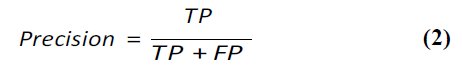


Recall measures the proportion of TP among all actual positives, and it is defined as mentioned in eq. (**3**).


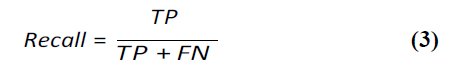


The F1 score represents the harmonic mean of precision and recall, offering a balanced measure between the two. Mathematically, it can be computed as shared in eq. (**4**).


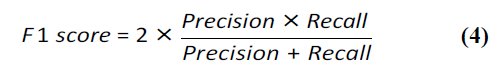


## 3 EXPERIMENTAL RESULTS

Results of multiple cerebral stroke-detecting classifiers are presented in this segment. ML models were prepared using Python 3.8-based Jupyter Notebook. The experiments were performed on a system equipped with an Intel Core i7 (Gen. 7) running in Windows 10 OS and powered by NVIDIA 1080 with 8 GB memory. Learning model effectiveness was evaluated by deploying multiple metrics.

### 3.1 ML Models Results by Eliminating Missing Values

This set of the experimental series was used to fix the missing values issue present in the dataset before applying ML models. Performance metrics of these models are outlined in Table **4**.

The findings reveal that SVM, LR, and RF attained the highest accuracy score of 98.14%. Regarding other evaluation parameters, RF and SVM achieved 98% precision, 100% recall, and 99% F1 score, while LR achieved 96% precision, 98% recall, and 97% F1 score. Following closely are KNN, ETC, with accuracies of 98.12% and 98.09%, respectively. Conversely, DT exhibited the weakest performance with an accuracy of 95.67%. Given the highly imbalanced nature of the dataset, the class-wise performance is significantly impacted. Across all classifiers, the performance for the stroke class is nearly zero, indicating performance enhancement of the model after eliminating the missing value from the dataset for the stroke class.

while Weighted Average accounts for class imbalance by weighting each class according to its frequency.

### 3.2 Results of ML Models using Mean Value Imputation

In the second set of experiments, the mean imputation technique is used for missing values, after which the resulting dataset is fed into the ML models. The outcomes of the learning models are presented in Table **5**.

while Weighted Average accounts for class imbalance by weighting each class according to its frequency.

The study’s results demonstrate that LR, SVM, and RF attained the highest accuracy values at 98.14%. These ML classifiers also achieved identical precision, recall, and F1 scores, all at 96%, 98%, and 97%, respectively. Furthermore, the results indicate an improvement in SGD’s performance following mean imputation, reaching 98.11%.

DT remains the least performing classifier with an accuracy score of 96.45%, showing a slight improvement over its previous performance.

### 3.3 Results of ML Models using KNN Imputation

Following mean imputation, we employed KNN imputation in this series of experiments. With the KNN imputer, all attribute instances were equalized to 43,400 each. However, despite this adjustment, the dataset remained imbalanced in this experimental set. Table **6** shows the results of the learning models.

The experiment outcomes suggest that RF, LR, and SVM achieved the maximum accuracy value of 98.14%. Given the dataset’s high imbalance, the performance of the stroke class was notably affected. There’s an observable improvement in KNN’s performance, reaching an accuracy score of 98.13%, putting it on par with the highest performers. ETC achieved an accuracy score of 98.07%. There were minor changes in the values of other parameters.

while Weighted Average accounts for class imbalance by weighting each class according to its frequency.

### 3.4 Results of ML Models Using SMOTE and KNN Imputer

Class imbalance and missing values issues were dealt with before feeding the dataset into the ML model, in this experimental series. The ML models’ performance on the updated dataset, using SMOTE and KNN imputer, is presented in Table **7**.

while Weighted Average accounts for class imbalance by weighting each class according to its frequency.

The results suggest that the ETC model has outperformed other learning models, accomplishing an accuracy score of 99.15% when using the KNN imputer and SMOTE datasets. It also attained recall, precision, and F1 scores of 99%. RF also demonstrated strong performance with a 98.94% accuracy score and identical F1 scores, precision, and recall of 99%. Moreover, there was an observed progress in the outcome of DT.

Conversely, the rest of the learning models experienced a degradation in performance, including LR, SVM, KNN, and SGD. SVM emerged as the least performing model with an accuracy score of 50.86%. This underscores the significance of imputation and class balancing methods on the results of learning models.

### 3.5 Results of ML Models using Mean Value Imputation and SMOTE

In the final set of experiments, mean imputation was used to address missing values. The class imbalance issue was tackled using SMOTE. The results of the ML models are presented in Table **8**.

The results of the ML models on the balanced dataset, without missing values using mean imputation, reveal that the tree-based model ETC performed exceptionally well, achieving an accuracy score of 99.25%, the highest among all sets of experiments. It also attained precision, recall, and F1 scores of 99%. Following closely is RF, which achieved an accuracy score of 99.02%. The class-wise accuracy achieved by ETC is also commendable compared to contemporary algorithms in this study. There’s an observed enhancement in the results of the learning models compared to scenario 4.

The performance of the SGD model in Table **8** shows an unusual pattern, where precision for the minority class, which is stroke, is relatively high, while recall and overall accuracy remain low. This behavior can be explained by the model’s tendency to predict fewer instances as the minority class. As a result, when a positive prediction is made, it is often correct, and the precision score is high, but many actual positive cases are missed, and the recall is low. Additionally, the model favors the majority class, resulting in higher recall for the non-stroke class. This trade-off shows the impact of class imbalance and highlights the importance of evaluating multiple performance metrics rather than relying only on accuracy.

while Weighted Average accounts for class imbalance by weighting each class according to its frequency.

### 3.6 Comparison of ML Models’ Results for All Scenarios

The effectiveness of the mean imputer is evaluated by us by comparing the results of ML prototypes with and without it. The results suggest that notable performance enhancement was observed in the ML models when SMOTE and the mean imputer were applied in the fifth experiment, as compared to other cases. Table **9** facilitates a comprehensive analysis of the performances in terms of accuracy and presents the results of the ML models for both circumstances.

### 3.7 K-fold Cross-validation Results

Models’ reliability is ensured using k-fold cross-validation, and results are provided in Table **10**. The results of five-fold cross-validation confirm the superiority of the recommended ETC models with SMOTE and mean imputation in terms of recall, accuracy, F1 score, and precision. Using this method, the ETC model achieved an accuracy of 0.9923, thus affirming the validity of the proposed system.

## 4 DISCUSSION WITH ADVANCED APPROACHES FROM LITERATURE

The performance of the recommended model is demonstrated by comparing it to the existing advanced models. Five accuracy-improving research-related modern models are chosen for the comparison. For example, [15] utilized DT for cerebral stroke detection, attaining an accuracy of 98.17%. In a different study [18], a two-class boosted DT with 6 features, and SMOTE was employed, gaining an accuracy of 96.8%. Likewise, [19] applied a deep neural network (DNN) with AutoHPO and RF regressor for imputation, attaining a 71.6% accuracy. The performance comparison between the existing and suggested models is presented in Table **11**.

The experimental results demonstrate that the proposed framework achieves high predictive performance for cerebral stroke detection, with the Extra Trees Classifier (ETC) combined with mean imputation and SMOTE yielding the best results. The improvement in accuracy indicates that addressing data quality issues, particularly missing values and class imbalance, plays a critical role in enhancing model reliability.

The superior performance of the ETC model can be attributed to its ensemble-based structure, which effectively captures complex relationships within clinical data while reducing overfitting. Compared to other machine learning models such as Random Forest, Support Vector Machine (SVM), and Decision Tree, ETC shows more stable and consistent performance across evaluation metrics, including precision, recall, and F1-score.

The application of SMOTE significantly improved the model’s ability to correctly classify minority class instances, which is essential in medical datasets where stroke cases are often underrepresented. Similarly, mean imputation helped in handling missing values without introducing significant bias, thereby improving data consistency and model performance.

When compared with existing studies, the results of this work are consistent with prior research demonstrating the effectiveness of ensemble learning methods in medical prediction tasks. However, many existing approaches rely on single-modality data, limiting their predictive capability. The highest accuracy of 99.25% was achieved using the Extra Trees Classifier with mean imputation and SMOTE. The 5-fold cross-validation accuracy of 99.23% indicates consistent model performance across different data splits. The slight variation between these values is due to differences between single train-test evaluation and cross-validation procedures.

From a clinical perspective, the proposed framework can support early stroke risk assessment and assist healthcare professionals in making timely decisions. Early prediction is crucial for reducing mortality and long-term disability associated with stroke, and AI-based tools can play an important role in improving preventive healthcare strategies.

## CONCLUSION

Cerebral stroke stands as a significant health threat, claiming the lives of millions annually; however, early detection and prompt intervention hold the potential to mitigate fatality risks. Despite the existence of automated models, challenges such as imbalanced class distributions and missing values present formidable obstacles to the generalizability of these models. In response, this research work makes use of mean data imputation with a SMOTE upsampled dataset. The results reveal that the extra tree classifier (ETC) model consistently exhibits robust performance across diverse datasets. In the original dataset, the model demonstrates remarkable performance in addressing class imbalance issues by giving accurate results. Moreover, when applied to the upsampled SMOTE dataset, the ETC model outperforms by handling unbalanced data, giving promising and reliable results. Furthermore, mean data imputation underscores the model adaptability in mitigating data preprocessing challenges. This research offers valuable insights for health practitioners seeking robust solutions in ML applications. While the proposed approach demonstrates strong predictive performance, several limitations should be acknowledged. The study relies on a single publicly available dataset, which may introduce data source bias and limit population diversity. Furthermore, the model has not been validated on external datasets, which raises its concerns about its generalizability across different clinical settings. In addition, practical deployment in healthcare environments may face challenges such as data variability, system integration, and the need for prospective validation. Future work will focus on addressing these limitations by incorporating multi-center datasets and validating the model in real-world clinical scenarios.

## Figures and Tables

**Fig. (1):**
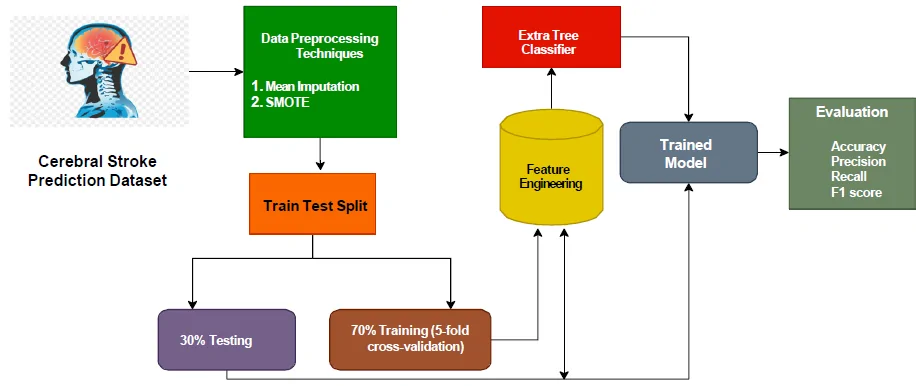
Proposed cerebral stroke prediction diagram.

**Table 1:** Summary of the related work.

**Ref.**	**Classifiers**	**Dataset Used**	**Performance**	**Class Balancing Technique**
[9]	RF, DT, VC, LR	Kaggle 5110 instances	96% RF	SMOTE
[10]	NB, KNN, LR, SGD, MLP, DT, RF, and Stacking	Kaggle 3254 instances	98% Stacking	SMOTE
[11]	RF, LR, SVM, KNN	Kaggle 5110 instances	95.5% RF and LR	Deleting missing values
[12]	RF, SVM, LR, KNN, NB, XGB	Kaggle 5110 instances	90.36% RF	SMOTE
[13]	KNN, LR, SVM, DT, RF, XGB, stacking, ANN	Kaggle 5110 instances	99% Stacking	SMOTE, SMOTE, ENN
[14]	RF, LR, KNN	Kaggle 43,400 instances	99% ADASYN+RF	ADASYN, SMOTE, ROSE
[15]	DT	Kaggle 43,400 instances	98.17%	

**Abbrevitaions**: *RF: Random Forest; DT: Decision Tree; LR: Logistic Regression; KNN: K-Nearest Neighbors; NB: Naïve Bayes; XGB: Extreme Gradient Boosting; SMOTE: Synthetic Minority Over-sampling Technique; ADASYN: Adaptive Synthetic Sampling.

**Table 2:** Dataset description.

**Attribute**	**Attribute Description**	**Variable Type**
Id	It contains the patient ID.	Continuous
Age	It contains the age of the patient.	Continuous
Gender	It represents the gender of the patient.	Categorical
Hypertension	It represents 0 for no hypertension and 1 for hypertension.	Categorical
Heart disease	It represents 0 for no heart disease and 1 for heart disease.	Categorical
Ever married	It contains the information about the patient, either it married or not.	Categorical
Work type	It contains information about the patient’s occupation.	Categorical
Residence type	It had the information about the urban/rural area of residence	Categorical
Avg glucose level	It shows the post-meal average glucose level.	Continuous
BMI	It represents the body mass index of the patient.	Continuous
Smoking status	It had the information about the patient’s smoking status.	Categorical
Stroke (target class)	It had patient information about stroke.	0 no stroke, 1 stroke

**Table 3:** Total values after imputation.

**Attribute**	**Total Values after Imputation**
Id	43400
Age	43400
Gender	43400
Hypertension	43400
Avg glucose level	43400
Stroke	43400
BMI	43400
Residence type	43400
Heart disease	43400
Work type	43400
Smoking status	43400
Ever married	43400

**Note:** *After imputation, all attributes contain complete records with no missing values.

**Table 4:** Performance of ML models after handling missing values in the dataset.

**Model**	**Accuracy**	**Class**	**Precision**	**Recall**	**F1 score**
DT	0.9566	1	0.05	0.07	0.06
		0	0.98	0.97	0.98
		Macro Average	0.52	0.52	0.52
		Weighted Average	0.97	0.96	0.96
RF	0.9814	1	0.00	0.00	0.00
		0	0.98	1.00	0.99
		Macro Average	0.49	0.50	0.50
		Weighted Average	0.96	0.98	0.97
SGD	0.9800	1	0.00	0.00	0.00
		0	0.98	1.00	0.99
		Macro Average	0.49	0.50	0.49
		Weighted Average	0.96	0.98	0.97
ETC	0.9809	1	0.00	0.00	0.00
		0	0.98	1.00	0.99
		Macro Average	0.49	0.50	0.50
		Weighted Average	0.96	0.98	0.97
KNN	0.9812	1	0.00	0.00	0.00
		0	0.98	1.00	0.99
		Macro Average	0.49	0.50	0.50
		Weighted Average	0.96	0.98	0.97
SVM	0.9814	1	0.00	0.00	0.00
		0	0.98	1.00	0.99
		Macro Average	0.49	0.50	0.50
		Weighted Average	0.96	0.98	0.97
LR	0.9814	1	0.00	0.00	0.00
		0	0.98	1.00	0.99
		Macro Average	0.49	0.50	0.50
		Weighted Average	0.96	0.98	0.97

**Note:** *Class 1 represents stroke cases, and Class 0 represents non-stroke cases. Macro Average refers to the unweighted mean of class-wise metrics.

**Table 5:** Performance of ML models using mean value imputation.

**Model**	**Accuracy**	**Class**	**Precision**	**Recall**	**F1 score**
DT	0.9645	1	0.08	0.09	0.08
		0	0.98	0.98	0.98
		Macro Average	0.53	0.53	0.53
		Weighted Average	0.97	0.96	0.97
RF	0.9814	1	0.00	0.00	0.00
		0	0.98	1.00	0.99
		Macro Average	0.49	0.50	0.50
		Weighted Average	0.96	0.98	0.97
SGD	0.9811	1	0.00	0.00	0.00
		0	0.98	1.00	0.99
		Macro Average	0.49	0.50	0.50
		Weighted Average	0.96	0.98	0.97
ETC	0.9807	1	0.00	0.00	0.00
		0	0.98	1.00	0.99
		Macro Average	0.49	0.50	0.50
		Weighted Average	0.96	0.98	0.97
KNN	0.9812	1	0.25	0.01	0.01
		0	0.98	1.00	0.99
		Macro Average	0.62	0.50	0.50
		Weighted Average	0.97	0.98	0.97
SVM	0.9814	1	0.00	0.00	0.00
		0	0.98	1.00	0.99
		Macro Average	0.49	0.50	0.50
		Weighted Average	0.96	0.98	0.97
LR	0.9814	1	0.00	0.00	0.00
		0	0.98	1.00	0.99
		Macro Average	0.49	0.50	0.50
		Weighted Average	0.96	0.98	0.97

**Note:** *Class 1 represents stroke cases, and Class 0 represents non-stroke cases. Macro Average refers to the unweighted mean of class-wise metrics.

**Table 6:** Performance of ML models using KNN imputation.

**Model**	**Accuracy**	**Class**	**Precision**	**Recall**	**F1 score**
DT	0.9637	1	0.05	0.05	0.05
		0	0.98	0.98	0.98
		Macro Average	0.51	0.52	0.51
		Weighted Average	0.96	0.96	0.96
RF	0.9814	1	0.00	0.00	0.00
		0	0.98	1.00	0.99
		Macro Average	0.49	0.50	0.50
		Weighted Average	0.96	0.98	0.97
SGD	0.9801	1	0.08	0.01	0.01
		0	0.98	1.00	0.99
		Macro Average	0.53	0.50	0.50
		Weighted Average	0.96	0.98	0.97
ETC	0.9807	1	0.00	0.00	0.00
		0	0.98	1.00	0.99
		Macro Average	0.49	0.50	0.50
		Weighted Average	0.96	0.98	0.97
KNN	0.9813	1	0.00	0.00	0.00
		0	0.98	1.00	0.99
		Macro Average	0.49	0.50	0.50
		Weighted Average	0.96	0.98	0.97
SVM	0.9814	1	0.00	0.00	0.00
		0	0.98	1.00	0.99
		Macro Average	0.49	0.50	0.50
		Weighted Average	0.96	0.98	0.97
LR	0.9814	1	0.00	0.00	0.00
		0	0.98	1.00	0.99
		Macro Average	0.49	0.50	0.50
		Weighted Average	0.96	0.98	0.97

Note: *Class 1 represents stroke cases, and Class 0 represents non-stroke cases. Macro Average refers to the unweighted mean of class-wise metrics.

**Table 7:** Performance of ML models using KNN imputation and SMOTE.

**Model**	**Accuracy**	**Class**	**Precision**	**Recall**	**F1 score**
DT	0.9754	1	0.97	0.98	0.98
		0	0.98	0.97	0.98
		Macro Average	0.98	0.98	0.98
		Weighted Average	0.98	0.98	0.98
RF	0.9894	1	1.00	0.98	0.99
		0	0.98	1.00	0.99
		Macro Average	0.99	0.99	0.99
		Weighted Average	0.99	0.99	0.99
SGD	0.5555	1	0.53	1.00	0.69
		0	0.97	0.11	0.20
		Macro Average	0.75	0.56	0.45
		Weighted Average	0.75	0.56	0.45
ETC	0.9915	1	1.00	0.98	0.99
		0	0.98	1.00	0.99
		Macro Average	0.99	0.99	0.99
		Weighted Average	0.99	0.99	0.99
KNN	0.9791	1	0.83	0.96	0.89
		0	0.95	0.80	0.87
		Macro Average	0.89	0.88	0.88
		Weighted Average	0.89	0.88	0.88
SVM	0.5086	1	0.51	0.72	0.60
		0	0.51	0.29	0.37
		Macro Average	0.51	0.51	0.48
		Weighted Average	0.51	0.51	0.48
LR	0.7927	1	0.77	0.84	0.80
		0	0.82	0.75	0.78
		Macro Average	0.79	0.79	0.79
		Weighted Average	0.79	0.79	0.79

**Note:** *Class 1 represents stroke cases, and Class 0 represents non-stroke cases. Macro Average refers to the unweighted mean of class-wise metrics.

**Table 8:** Performance of ML models using mean imputation and SMOTE.

**Model**	**Accuracy**	**Class**	**Precision**	**Recall**	**F1 score**
DT	0.9785	1	0.98	0.98	0.98
		0	0.98	0.98	0.98
		Macro Average	0.98	0.98	0.98
		Weighted Average	0.98	0.98	0.98
RF	0.9902	1	1.00	0.98	0.99
		0	0.98	1.00	0.99
		Macro Average	0.99	0.99	0.99
		Weighted Average	0.99	0.99	0.99
SGD	0.5831	1	0.71	0.28	0.40
		0	0.55	0.89	0.68
		Macro Average	0.63	0.58	0.54
		Weighted Average	0.63	0.58	0.54
ETC	0.9925	1	1.00	0.99	0.99
		0	0.99	1.00	0.99
		Macro Average	0.99	0.99	0.99
		Weighted Average	0.99	0.99	0.99
KNN	0.8435	1	0.79	0.95	0.86
		0	0.93	0.74	0.83
		Macro Average	0.86	0.84	0.84
		Weighted Average	0.86	0.84	0.84
SVM	0.6030	1	0.72	0.34	0.46
		0	0.57	0.87	0.69
		Macro Average	0.64	0.60	0.57
		Weighted Average	0.64	0.60	0.57
LR	0.7898	1	0.77	0.83	0.80
		0	0.82	0.75	0.78
		Macro Average	0.79	0.79	0.79
		Weighted Average	0.79	0.79	0.79

**Note:** *Class 1 represents stroke cases, and Class 0 represents non-stroke cases. Macro Average refers to the unweighted mean of class-wise metrics.

**Table 9:** Comparative performance of ML models under different preprocessing scenarios in terms of accuracy for cerebral stroke prediction.

**Model**	**Missing Value Removal**	**Mean** **Imputation**	**KNN** **Imputation**	**KNN Imputation + SMOTE**	**Mean Imputation** **+ SMOTE**
DT	0.9567	0.9645	0.9637	0.9754	0.9785
RF	0.9814	0.9814	0.9814	0.9894	0.9902
SGD	0.9800	0.9811	0.9801	0.5555	0.5831
ETC	0.9809	0.9807	0.9807	0.9915	0.9925
KNN	0.9812	0.9812	0.9813	0.9791	0.8435
SVM	0.9814	0.9814	0.9814	0.5086	0.6030
LR	0.9814	0.9814	0.9814	0.7927	0.7898

**Note:** *All performance metrics (Accuracy, Precision, Recall, and F1-score) are reported in percentage (%). The results are obtained under different preprocessing scenarios, including missing value removal, mean imputation, KNN imputation, and SMOTE-based class balancing.

**Table 10:** 5-fold cross-validation results of the ETC.

**Number of Folds**	**Achieved Accuracy**
1st Fold	0.9927
2nd Fold	0.9935
3rd Fold	0.9918
4th fold	0.9911
5th Fold	0.9924
Average	0.9923

**Table 11:** Comparison with other studies.

**Reference**	**Classifiers**	**Performance (Accuracy)**
[15]	DT	98.17%
[18]	LR	73.52%
[19]	PCA-Kmeans + DNN + Focal Loss	92%
Proposed	ETC with Mean Imputation and SMOTE	99.25%

## Data Availability

The dataset is publicly available at this link: https://www.kaggle.com/datasets/shashwatwork/cerebral-stroke-predictionimbalaced-dataset.

## LIST OF ABBREVIATIONS

AI: Artificial Intelligence
CNN: Convolutional Neural Network
MRI: Magnetic Resonance Imaging
CT: Computed Tomography
KNN: K-nearest Neighbor
RF: Random Forest
DT: Decision Tree
SVM: Support Vector Machine
VC: Voting Classifier
AUC: Area Under Curve
ML: Machine Learning
DL: Deep Learning
SMOTE: Synthetic Minority Over-sampling Technique
XAI: Explainable Artificial Intelligence
TP: True Positive
TN: True Negative
FP: False Positive
FN: False Negative
